# Histopathologically Confirmed Nuck’s Cyst Mimicking Lymphadenopathy in a Young Female: A Case Report

**DOI:** 10.7759/cureus.87621

**Published:** 2025-07-09

**Authors:** Andrea M Salas Carlock, Jose M Zepeda Torres, Martin Islas Torres, Erik D Palomares Castillo, Héctor A Benítez Jauregui, Rodrigo Hernández Ramírez, Maria A Lastra Santiago, Herbert I Cáceres Espindola, Angeles del Carmen Rabago Moreno, Alberto Sánchez Navarro Bermejo, Pedro Cardenas Cruz

**Affiliations:** 1 General Surgery, Centro Médico Nacional de Occidente, Instituto Mexicano del Seguro Social, Guadalajara, MEX; 2 Surgery, Centro Médico Nacional de Occidente, Instituto Mexicano del Seguro Social, Guadalajara, MEX

**Keywords:** hydrocele of the canal of nuck, nuck's canal, nuck's cyst, surgical case reports, the canal of nuck

## Abstract

Hydrocele of the canal of Nuck is an exceedingly rare congenital anomaly in females, often mimicking inguinal hernias and frequently misdiagnosed due to limited awareness. This report presents a 26-year-old woman with cyclical inguinal swelling, ultimately diagnosed intraoperatively as a Nuck's cyst, confirmed by histopathology revealing endometrial tissue within the cyst. The condition arises from persistent communication between the peritoneal cavity and the inguinal canal along the round ligament, leading to cyst formation. Surgical excision remains the definitive treatment, with approaches tailored according to cyst type and extent - open or laparoscopic - with additional repair of inguinal defects, as necessary. Accurate preoperative imaging and awareness are essential to differentiate Nuck's cyst from other inguinal masses, ensuring optimal outcomes and preventing complications such as herniation or torsion. This case underscores the importance of considering Nuck’s cyst in the differential diagnosis of inguinal lesions in women and highlights the surgical principles for effective management.

## Introduction

Hydrocele of the canal of Nuck is an exceedingly rare congenital condition resulting from the incomplete obliteration of the processus vaginalis in females. Its prevalence among females aged 0 to 16 years is approximately 0.76%, according to the World Health Organization. While typically presenting in pediatric populations, reports of Nuck’s canal cysts in adult women remain scarce, limited primarily to isolated case reports. To date, approximately 50 publications indexed in PubMed have documented this condition in adult females. This case is particularly noteworthy because it involves a young female patient - a demographic less frequently reported in the literature - and was initially suspected of lymphadenopathy. We believe this case warrants publication to contribute to the limited existing knowledge, provide insights into differential diagnosis in young females, and highlight the importance of considering Nuck’s cyst in the differential diagnosis of groin masses beyond the pediatric age group [1].

Due to a general lack of awareness among frontline clinicians regarding the diagnosis and management of this condition, misdiagnosis and inadequate treatment are common. To illustrate this, we present a noteworthy case involving a young female patient.

## Case presentation

**Figure 1:**
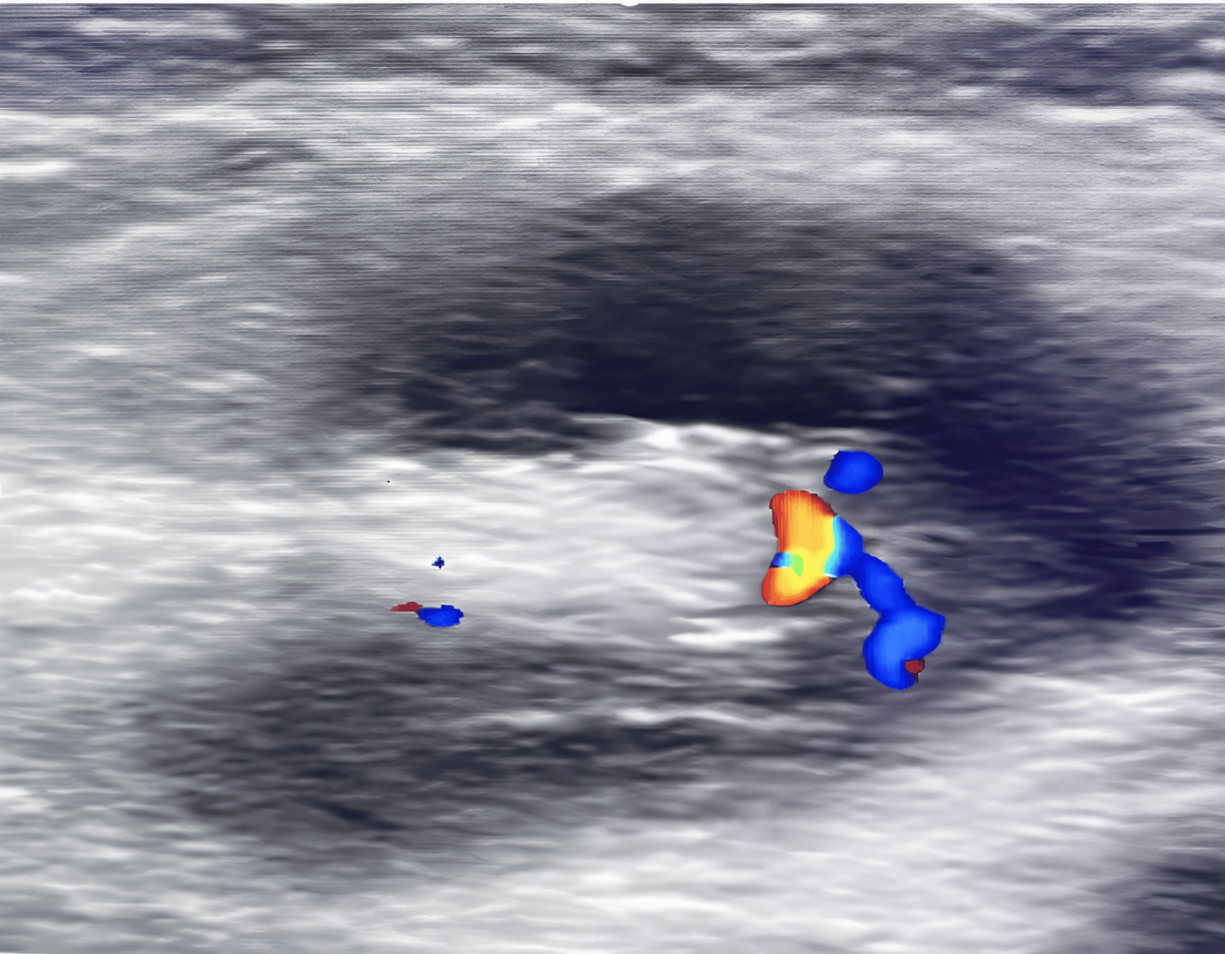
Inflammatory-appearing lymph nodes measuring 8 × 4 mm and 10 × 6 mm

A 26-year-old female patient presented with a palpable lesion in the right inguinal region. The lesion exhibited intermittent fluctuations in size that correlated with the menstrual cycle. Physical examination revealed a 2 × 2 cm indurated, firm mass, fixed to the deep tissues and tender on palpation. After six months of progressive symptoms and lesion growth, an ultrasound was performed (Figure 1), identifying two inflammatory-appearing lymph nodes measuring 8 × 4 mm and 10 × 6 mm; no hernial defects were observed.

**Figure 2:**
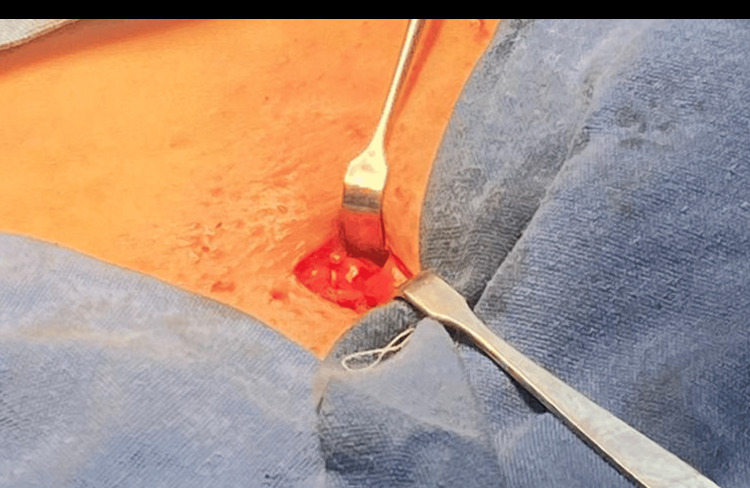
Violet lesion originating from the inguinal canal consistent with a Nuck’s cyst

Due to persistent discomfort associated with the increasing volume, an excisional biopsy of the lymphadenopathy was scheduled. During the procedure, a right inguinal incision was made. A vesicular, violet lesion originating from the inguinal canal was encountered (Figure 2). The lesion was dissected away from the round ligament, with spontaneous opening and extrusion of a citrine fluid upon manipulation. A peritoneal window was created, and the deep inguinal ring was explored, revealing no communication with the peritoneal cavity.

Histopathological analysis confirmed the diagnosis of a Nuck's cyst, with a capsule and the presence of an endometrial tissue focus. At six-month follow-up, the patient remained asymptomatic, with no apparent complications.

## Discussion

Clinically, it closely resembles an inguinal hernia, typically presenting as a mass extending from the inguinal region to the labia majora. The underlying mechanism involves a pouch-like protrusion formed when intraperitoneal fluid enters the parietal peritoneum and descends along the inguinal canal through the inguinal ring, following the path of the round ligament of the uterus. Consequently, it is sometimes referred to as a “female hydrocele” [1,2].

In cases with notable symptoms - such as significant swelling, rapid growth, or suspicious findings on auxiliary examinations - surgical intervention is recommended. Conversely, if the lesion is asymptomatic or only mildly enlarging, there is a risk that traction on the round ligament could cause the ovary and fallopian tube to protrude into the abdominal cavity, potentially leading to an indirect inguinal hernia, torsion, or necrosis of the ovarian broad ligament [1].

The surgical management of a Nuck's cyst (hydrocele of the canal of Nuck) in a young female patient - particularly when it mimics lymphadenopathy, as in our case - requires complete excision of the cystic lesion, with the surgical approach tailored to the anatomical type and extent of the cyst. Preoperative imaging with ultrasound and/or magnetic resonance imaging is essential for diagnosis and surgical planning, as these cysts are often misdiagnosed due to their rarity and nonspecific presentation [1,2].

For superficial (external, Type 1) Nuck's cysts, an open inguinal approach is generally recommended, allowing for direct visualization and excision of the cyst. If the cyst is intra-abdominal (Type 2) or has a more complex configuration (Type 3), a laparoscopic approach may be preferred, as it facilitates both cyst excision and assessment of the inguinal canal and peritoneal cavity [1-3]. In cases where the cyst is adherent or difficult to dissect laparoscopically, a combined approach with a small auxiliary inguinal incision may be necessary to ensure complete removal while preserving the round ligament of the uterus [1].

If the presence of the cyst has resulted in widening of the inguinal canal or is associated with a hernia, concurrent hernioplasty with mesh reinforcement should be considered to prevent recurrence or future herniation [1,2]. Closure of the internal inguinal ring is also recommended if a defect is identified intraoperatively [3,4].

Histopathological examination of the excised specimen is required to confirm the diagnosis and exclude other pathologies, such as endometriosis or, rarely, ectopic pregnancy within the cyst [3,4]. Postoperative outcomes are generally favorable, with low recurrence rates when complete excision and appropriate repair are performed [1,2,5].

In summary, the standard of care is surgical excision of the Nuck's cyst, with the approach (open vs. laparoscopic) individualized based on cyst type and anatomical considerations, and with concurrent hernia repair, as indicated [1-3].

## Conclusions

Nuck’s cyst, though rare in adult women, should be included in the differential diagnosis when evaluating inguinal masses, especially in patients presenting with cystic features or cyclical changes. The diagnosis of this condition often relies on clinical suspicion supported by appropriate imaging; however, the specific diagnostic approaches, and whether they were utilized in this case, are not elaborated upon in the case presentation or discussion. The choice of surgical management - whether open or laparoscopic - depends on the cyst’s characteristics and extent, along with the presence of any inguinal canal defects, and should aim to prevent recurrence and complications. Histopathological examination plays a vital role in confirming the diagnosis and ruling out other pathologies, such as endometriosis. Recognizing and managing Nuck’s cysts in a timely manner can lead to favorable patient outcomes, reducing the risk of misdiagnosis and ensuring complete resolution of symptoms.
